# A childhood immunization education program for parents delivered during late pregnancy and one-month postpartum: a randomized controlled trial

**DOI:** 10.1186/s12913-019-4622-z

**Published:** 2019-11-05

**Authors:** Hiroko Otsuka-Ono, Narumi Hori, Hiroshi Ohta, Yukari Uemura, Kiyoko Kamibeppu

**Affiliations:** 1 Department of Family Nursing, Graduate School of Medicine, The University of Tokyo, 7-3-1 Hongo, Bunkyo, Tokyo, 113-0033 Japan; 2 Disease Control and Prevention Center, National Center for Global Health and Medicine, Tokyo, Japan; 3 Seto Hospital, Saitama, Japan; 4 Biostatistics Division, Central Coordinating Unit, Clinical Research Support Center, The University of Tokyo Hospital, Tokyo, Japan

**Keywords:** Education, Childhood immunization, Parents, Decision-making, Immunization rate

## Abstract

**Background:**

Improved immunization rates have reduced the incidence of vaccine-preventable diseases (VPDs) in advanced nations. Japan’s unique vaccination system classifies vaccines into routine vaccines ostensibly required under the Preventive Vaccination Law and recommended but optional vaccines, although all vaccines are in fact voluntary. In Japan, low immunization rates, particularly for optional vaccines, have resulted in high rates of sequelae and death. The decision as to whether a child will receive a vaccine depends on the parents, who must obtain information, make inquiries, and make the required payment, the last of which is a major barrier. This randomized, controlled trial was conducted to evaluate the effectiveness of an immunization education program designed to meet mothers’ needs.

**Methods:**

This randomized controlled trial assigned pregnant women to intervention or control groups. The intervention was individual education sessions involving the children’s fathers in shared decision-making on whether or not to immunize their child. A survey was conducted before and after the intervention. Data were analyzed using the intention-to-treat principle.

**Results:**

Of 225 pregnant women, 175 (78%) participated and 171 replied to the post-survey. At age 3 months, intervention infants had higher self-reported immunization rates for hepatitis B virus vaccine (76% vs. 49%; *P* < 0.001) and rotavirus vaccine (84% vs. 68%; *P* = 0.019) than control group infants. The percentage of parents intending to vaccinate their infants was higher in the intervention group (77% vs. 52%; *P* < 0.01). Improvements in scores for basic knowledge (mean [SD]: 5.5 [3.6] vs. 3.0 [3.8], range: 10–30; *P* < 0.001), advanced knowledge (mean [SD]: 5.1 [2.4] vs. 2.8 [2.5], range: 5–15; *P* < 0.001), and health literacy regarding immunization (mean [SD]: 0.5 [0.8] vs. 0.2 [0.6], range: 1–5; *P* < 0.01) were higher in the intervention group. The rate of decision making by both parents (68% vs. 52%; *P* < 0.05) was higher in the intervention group.

**Conclusions:**

Our findings confirmed the program’s effectiveness. The intervention improved immunization rates, the percentage of parents intending to vaccinate their infants and knowledge scores. Interventions which directly and indirectly involved fathers in shared decision-making on whether to immunize their child were effective, as were individualized interventions that provided parents with access to up-to-date information.

**Trial registration:**

UMIN000012575. Registered 14 December 2013 (The study was prospectively registered).

## Background

Improved immunization rates have reduced the incidence of vaccine-preventable diseases (VPDs) in advanced nations [1, 2]. Japan has a unique vaccination system which consists of routine vaccines required under the Preventive Vaccination Law [3] and optional vaccines which must be paid for by consumers. The current national immunization program includes only nine routine vaccines (for twelve targeted diseases), while all other vaccines are optional. As a consequence, vaccination rates for optional vaccines have remained low, and the incidence of the target diseases has remained high. The initial immunization law established in 1948 considered receiving vaccines a duty, and penalties were imposed on noncompliant citizens. However, the occurrence of adverse effects resulting from vaccines in the 1980s and 90s, and emphasis of the risks of vaccination by the media planted doubts among civilians. In 1994, the immunization law was revised, and immunization was changed from a civic “duty” to an “effort duty” [4]. Since 1994, all vaccinations, including those ostensibly required under law, have been voluntary.

In Japan, low immunization rates, particularly for optional vaccines, have resulted in high rates of sequelae and death [5–8]. When the present survey was conducted, a 2-month-old child may have received the *Haemophilus influenzae* type b vaccine (Hib) and 13-valent pneumococcal conjugate vaccine (PCV13), which are routine, but may have delayed receipt of hepatitis B and rotavirus vaccines, because they are optional and costly (about USD 50–150 each). The hepatitis B vaccine became a routine vaccine for infants (less than 1 years old) in Japan in October 2016, but remains an optional vaccine for all other citizens. Hepatitis B is a severe VPD, with 80–90% of infants infected with the hepatitis B virus (HBV) during the first year of life and 30–50% infected before the age of 6 years developing chronic infection [9]. The decision as to whether a child will receive a vaccine depends on the parents, who need to obtain information, make inquiries, and make the required payment, the last of which is a major barrier. If parents do not take these actions, their children will not be vaccinated for optional vaccines.

Apart from cost, the information provided to parents about optional vaccinations is insufficient compared to that on routine vaccines [10]. Parents receive information on vaccinations through pamphlets [11], which are distributed by local governments within 2 months of childbirth. However, this information may not be sufficient for parents to make informed decisions on childhood optional vaccinations. Further, although education programs for parturients regarding immunization are being introduced in some advanced medical institutions, the contents and methods of the education vary by institution. This lack or inconsistency in information provided may cause parents to regard optional vaccines as being of low importance [12]. In addition, the misconception that infection provides better immunity than vaccination may also reduce vaccination rates [13].

According to a Cochrane review, randomized controlled trials of face-to-face interventions for educating parents on childhood vaccination have yielded limited evidence of low quality, and their effect could not be determined [14]. In addition, no data were available on the secondary outcomes of interest [14]. Although improving health literacy for immunization has attracted substantial attention [15], no educational program to improve the general health literacy of parents has been developed. Educational intervention in pregnant women has improved optional vaccination rates for HBV for infants, increased the intention of mothers to vaccinate infants [16, 17], and increased the knowledge of mothers regarding vaccination [16, 17]. To our knowledge, however, no studies have demonstrated the effectiveness of an educational intervention program for routine and optional vaccines designed to meet mothers’ needs and which require direct and indirect involvement of children’s fathers. Partners (children’s fathers) who attend prenatal care can work directly with medical professionals to share information and engage in joint decision-making with mothers about whether or not to immunize their child. In cases where the partner does not attend, medical professionals can encourage the mother to share information with the partner to aid in promoting discussions regarding vaccinations. In this way, advice from medical professionals can indirectly reach the partner.

Accordingly, we undertook a study to develop a suitable educational intervention program. Mothers’ needs were defined in this study as the information needs of mothers with young children who require information about immunization. We then developed a guidebook and a checklist based on pretest interviews with mothers of small children. This process revealed that mothers needed to involve the children’s fathers (partners) in shared decision-making on whether or not to immunize their child.

Here, we evaluated the effectiveness of this immunization education program designed to meet mothers’ needs in Japan, where some vaccinations are optional.

## Methods

The outline of the study is presented in Additional file 1: Figure S1.

### Study design

A randomized parallel-group trial.

### Study setting

Participants were recruited between December 18, 2013 and February 20, 2014 at a private hospital outside Tokyo which provides advanced care for childhood immunization. As part of the standard care provided by this hospital, all participants received group guidance on immunization after delivery.

### Eligibility and enrollment

Pregnant women aged 18 years or older who were not scheduled to change hospital were recruited during gestational weeks 29–33. Participants signed a consent form and completed a baseline survey. Data were then collected using a self-administered survey. Random allocation was conducted by a central registration system of the JCRAC Data Center, Department of Clinical Study and Informatics, Center for Clinical Sciences, National Center for Global Health and Medicine. Each participant received an identification number and was randomly assigned to an intervention or control group by the central registration system, where they were stratified based on ‘primipara’ or ‘para’ status. Participants were blinded to their group assignment at recruitment and during completion of the baseline questionnaire. As the intervention was an educational program, blinding of study participants was not possible after groups were assigned. The pediatrician or pharmacist who provided group guidance was blinded to the status of each group. The obstetricians and pediatricians, nursing staff, and hospital staff who might also recommend vaccination were also blinded to participants’ group status. The person who conducted the data analysis was blinded to group participation. This study was approved by the Institutional Review Board of the University of Tokyo.

### Intervention

In addition to the group guidance regarding immunization provided by the hospital, participants in the intervention group also received two individual immunization education sessions, once during late pregnancy and the second at the one-month postpartum check-up. The individual education sessions lasted approximately 10 min during late pregnancy and 3–5 min at the one-month postpartum check-up. The first intervention session used the guidebook with an infant immunization schedule [18, 19]. Participants assigned to the intervention group were provided with the guidebook and infant immunization schedule prior to the intervention after group assignment so that they could read them during the waiting time for the prenatal checkup. The second intervention consisted of a check-up to determine whether parents had sought a pediatrician or primary care physician to vaccinate their child and confirmation of the date of initial vaccination using the checklist. When possible, the children’s fathers and the women’s partners or family members also attended the two sessions, which were conducted in an outpatient setting by a single investigator. The post-survey was mailed to all participants approximately 100 days after delivery. Participants who did not reply within 10 days of mailing the post-survey were sent a reminder in the form of a postcard in the mail.

### Development and contents of intervention

The immunization education program was developed following focus group interviews and individual interviews with mothers of small children. The immunization education program we developed was based around four elements, namely the participatory approach of fathers or family members, using a guidebook as a communication tool to share information and aid in promoting discussions regarding vaccinations; encouraging parents to seek a pediatrician or primary-care physician (doctor’s clinic or healthcare facility) through both conversation and handouts before delivery to vaccinate their child; communicating the concept of VPDs, timing of initial vaccinations, and the importance of vaccination timeliness; and providing each expectant parent with easy access to up-to-date information regarding immunization (via URLs, inquiries based on information provided by their local government through their website or public relations magazines, and home visits by public health workers to observe new babies).

### Development and contents of guidebook

The guidebook was developed based on previous studies [16, 17, 20], Vaccine Information Statements [21], reports [22], educational material [23, 24], and internet sites [2, 25, 26]. The contents of the guidebook also incorporated the opinions of experts (a pediatrician, gynecologist, nurse, midwife, nurse specialized in infectious diseases, clinical psychologist, and researcher). The main contents of the guidebook were: a list of diseases that can be prevented with vaccines, important information for scheduling vaccines, that the necessity of routine vaccinations and optional vaccinations are the same, simultaneous vaccinations, the beneficial and adverse effects of vaccinations, and vaccination at 2 months of age (optional vaccinations included those for hepatitis B and rotavirus at the time of the survey).

### Measures

After the survey items were examined by researchers, a pediatrician, midwives, nurses, a public health nurse, infectious disease professionals and psychology experts, we pretested them on pregnant women (primiparous and multipara) and mothers with infants. The items were subsequently modified to improve understanding. Questionnaire surveys were evaluated at baseline and at 3 months. Each questionnaire was completed in about 5–10 min. The primary outcome measure was the self-reported current immunization status for the HBV vaccine. We chose this primary outcome measure because hepatitis B is a severe VPD and has severe effects in children younger than 6 years of age. The secondary outcomes were immunization status for the rotavirus vaccine, Hib vaccine, and PCV13; intention to receive vaccines; parental discussion regarding vaccinations; joint parental decision-making on whether their child would be vaccinated; and seeking a pediatrician or primary-care physician to administer vaccinations. Additional secondary outcomes were changes in maternal knowledge, attitudes and beliefs, and health literacy as measured using the pre- and post-surveys.

The date of each vaccination was recorded. When an infant was not immunized, the scheduled date for each vaccination was recorded. The immunization status of infants was determined at 3 months of age. The 92-day assessment was used as the standard measure to time the initiation of immunization [27]. The immunization status of each infant was evaluated between 62 and 92 days of age because infant immunization begins at 2 months of age based on Japanese immunization law.

Intention to immunize was measured on a 4-point scale with scores of 1 (no intention), 2 (undecided), 3 (yes, for certain vaccines), and 4 (yes, for most vaccines). Parental discussion regarding vaccinations was measured for participants with a partner by asking the extent to which, “I think I can discuss vaccinating our child with my child’s father.” Responses were measured on a 4-point Likert scale ranging from 1 (strongly agree) to 4 (strongly disagree).

Decision-making regarding vaccinations was evaluated by asking, “Who made the decision on whether or not to vaccinate your child?” Participants selected from “Self and Child’s Father (Partner)”, “Self”, “Child’s Father (Partner)” or “Other”. The status of seeking a pediatrician or primary care physician to administer vaccinations was evaluated by asking, “Have you looked for a pediatrician to administer vaccinations?” with responses of “I have already sought one”, “I have not yet sought one”, “I have no intention of seeking one” or “I need not seek one” (e.g., when the mother already has a pediatrician or primary care physician to administer vaccinations).

Knowledge was measured on a 3-point Likert scale consisting of “I don’t think so”, “I don’t know” and “I think so”, and evaluated using scores of 1 (I don’t know), 2 (incorrect answer), or 3 (correct answer). Basic knowledge (Additional file 1: Table S1) consisted of 10 items and advanced knowledge (Additional file 1: Table S2) of 5 items, with higher scores indicating greater knowledge.

Attitudes and beliefs regarding VPDs and childhood vaccination were evaluated based on the Health Belief Model (HBM) [28] and the Integrated Behavioral Model (IBM) [29]. These models were used to assess psychosocial factors using a questionnaire [16, 30] based on 5-point Likert scales with responses ranging from 1 (strongly disagree) to 5 (strongly agree). The Japanese version of attitudes and beliefs regarding VPDs and childhood vaccination (Additional file 1: Table S4) has been shown to have the same level of internal consistency as the original version except for one item (self-efficacy) and acceptable levels of reliability and validity [31]. We modified the scale of health literacy [32] to evaluate health literacy regarding vaccination of children (Additional file 1: Table S3), with permission from the scale’s developer. Each item was rated on a 5-point scale, with potential responses ranging from 1, “strongly disagree” to 5, “strongly agree”. The mean score of the five items was used in the analyses, with higher scores indicating greater health literacy regarding immunization.

Demographic information was collected, including age, job status, date of birth of the infant, education, number of children, family structure, and annual income.

### Sample size

Sample size was calculated by assuming an HBV vaccination rate of 11.1% in the control group and 34.3% in the intervention group [16] with a statistical power of 80% and an adjusted type 1 error of 5%. This resulted in 57 participants in each study group being sufficient. However, to account for the likely exclusion of participants by assuming an expected response rate for the second questionnaire survey of approximately 65%, 175 (total) registered cases were considered necessary. All calculations were performed using G*Power software, version 3.1.7 (University Kiel, Kiel, Germany) [33].

### Analyses

Data were analyzed using the intention-to-treat principle [34, 35]. Descriptive statistics were used to assess distribution of the background and outcome variables of respondents. Fisher’s exact test was used to perform bivariate analyses and to examine data distributions and associations between groups. The Mann-Whitney U test was used to compare mean scores and measure secondary outcomes.

Spearman’s rank correlation coefficient was used to examine the association between parental discussion and decision-making regarding vaccinations. All *P*-values were two-tailed, and *P* < 0.05 was considered significant. The Statistical Package for Social Sciences (IBM SPSS, Armonk, NY, USA), Windows version 21.0, was used for analyses.

## Results

**Fig. 1:**
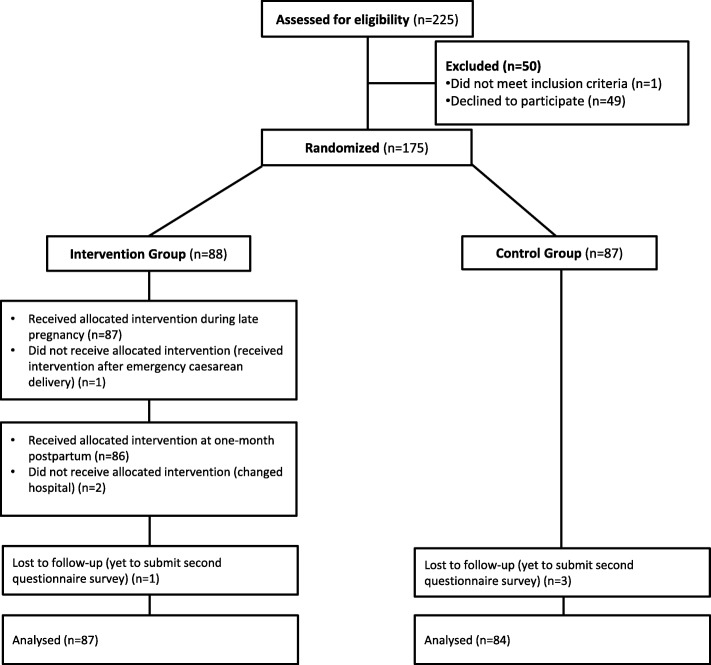
Flowchart of participants

Of 225 pregnant women, 175 (78%) participated in the study. In accordance with the randomization of data from the JCRAC Data Center, 88 women were allocated to the intervention group and 87 to the control group. A total of 171 post-survey questionnaires (98%) were returned. Figure 1 shows a detailed flow chart of participants in each group. Table 1 shows the characteristics of participants at baseline and between the two arms. In the intervention group, 85 of 88 women received interventions during late pregnancy and at one-month postpartum. One woman did not receive the allocated intervention during late pregnancy (but received the first intervention after delivery) due to emergency caesarean delivery, and two did not receive the allocated intervention at 1 month postpartum (only the intervention during late pregnancy) due to changing hospitals.

**Table 1:** Characteristics of study participants

Characteristic	Intervention (*n* = 87)	Control (*n* = 84)
Age, mean (standard deviation)		
Mother	32.8 (3.9)	33.0 (4.9)
Father	34.8 (4.9)	35.1 (5.8)
Delivery history, n (%)		
Primipara	46 (53%)	46 (55%)
Multipara	41 (47%)	38 (45%)
Marital status, n (%)		
Married	86 (99%)	83 (99%)
Unmarried couple	1 (1%)	1 (1%)
Mother’s highest education level completed, n (%)		
Middle/high school	9 (11%)	12 (14%)
Vocational school	21 (24%)	20 (24%)
Junior college	20 (23%)	20 (24%)
University	35 (40%)	30 (36%)
Graduate	2 (2%)	2 (2%)
Number of children, n (%)		
0	46 (53%)	46 (55%)
1	32 (37%)	30 (36%)
≥ 2	9 (10%)	8 (10%)
Job status, n (%)		
Mother		
Unemployed	38 (44%)	39 (46%)
Full-time job	35 (40%)	36 (43%)
Part-time job	14 (16%)	8 (10%)
Father		
Full-time job	84 (97%)	81 (96%)
Self-employed	3 (3%)	3 (4%)
Returned to work after maternity leave, n (%)	46 (53%)	46 (55%)
Annual income (thousand yen), n (%)		
< 2000	0	2 (2%)
2000–3999	10 (12%)	9 (11%)
4000–5999	35 (42%)	31 (37%)
6000–7999	17 (20%)	24 (29%)
8000–9999	16 (19%)	11 (13%)
≥ 10,000	6 (7%)	7 (8%)

Fifty (58%) women in the intervention group and 59 (70%) in the control group received group guidance (standard education). Twenty (23%) fathers (partners) and 2 (2%) participants’ mothers in the intervention group participated in the educational intervention during late pregnancy. The following relatives also took part in the intervention at 1 month postpartum: 30 (35%) fathers (partners), 19 (11%) mothers of the participants, 4 (2%) fathers of the participants, 2 (1%) mothers and fathers of the participants, and 1 (1%) older sister of a participant.

Table 2 shows the immunization status after the intervention. The overall infant immunization status for the four examined vaccines during the 92-day follow-up period was higher in the intervention group (72%) than in the control group (43%) (*P* < 0.001). On comparison of the rates of immunization for each vaccine, the rate for the HBV vaccine was higher in the intervention group (76%) than in the control group (49%) (*P* < 0.001), and that for the rotavirus vaccine was higher in the intervention group (84%) than in the control group (68%) (*P* = 0.019). In contrast, no significant differences between groups were seen in vaccination status for the Hib (*P* > 0.999) or PCV13 vaccines (*P* = 0.491).

**Table 2:** Immunization status self-reported of groups after intervention

Outcome	Intervention (*n* = 87)	Control (*n* = 84)	*P*-value
Vaccination^a^, n (%)			
Hepatitis B virus (HBV), mean (SD)	66 (76%)	41 (49%)	< 0.001^***^
Rotavirus	73 (84%)	57 (68%)	0.019^*^
*Haemophilus influenzae* type b (Hib)	83 (95%)	80 (95%)	> 0.999
Conjugated 13-valent pneumococcal (PCV13)	84 (97%)	79 (94%)	0.491
Number of completed vaccinations^b^ (range: 0–4)	3.5 (0.9%)	3.1 (1.0%)	< 0.001^***^
Completed four vaccinations^a^, n (%)	63 (72%)	36 (43%)	< 0.001^***^

^*^p < 0.05, ^***^p < 0.001

^a^Fisher’s exact test

^b^Mann-Whitney U test

Table 3 shows the decision-making status and participation rates of intervention during late pregnancy. Fifteen (75%) of 20 fathers (partners) participated in the intervention, with both parents involved in the decision-making. Even when only mothers participated in the intervention, both parents were involved in the decision-making in 42 (65%) of 65 cases. Table 3 shows that there were no significant differences in the decision-making status or participation rate for couples.

**Table 3:** Decision-making and participation rates in intervention during late pregnancy

Decision		Participation rate, n (%) (*n* = 85 total)		*P*-value
		Mother alone	Father and mother	
Person who decided whether or not child would be vaccinated	Father and mother	42 (65%)	15 (75%)	0.430
	Mother alone	23 (35%)	5 (25%)	

Fisher’s exact test

The rate of decision-making by both parents versus the mother alone was higher in the intervention group (*P* < 0.05), although the degree of parental discussion regarding vaccinations did not significantly differ (Table 4). Spearman’s rank correlation coefficient between parental discussion and decision-making regarding vaccinations was 0.5 (*P* < 0.001). No significant difference was noted between groups regarding seeking a pediatrician or primary care physician to vaccinate their child (*P* = 0.469) (Table 4).

**Table 4:** Comparison between groups for parental discussion regarding vaccinations, decision-making, and seeking a pediatrician for vaccinations

Outcome	Range	Intervention (*n* = 87)	Control (*n* = 84)	*P*-value
Parental discussion regarding vaccinations,^b^ mean (standard deviation)	1–4^c^	1.5 (0.6)	1.6 (0.7)	0.672
Decision-making regarding vaccinations,^a^ n (%)				
Who decided whether your child should receive vaccinations?				0.043^*^
Father and mother		59 (68%)	44 (52%)	
Mother		28 (32%)	40 (48%)	
Seeking a pediatrician for vaccinations,^a^ n (%)				
I have already sought a pediatrician for vaccinations		78 (90%)	68 (81%)	0.469
I have not yet sought a pediatrician for vaccinations		0 (0%)	1 (1%)	
I need not seek a pediatrician for vaccinations		9 (10%)	15 (18%)	

^*^p < 0.05

^a^Fisher’s exact test

^b^Mann-Whitney U test

^c^Lower scores indicate that the mother engaged in a greater degree of discussion with the child’s father regarding their child’s vaccinations

However, the groups did significantly differ with regard to intent to immunize infants (*P* = 0.001) (Table 5). The proportion of participants who answered “yes” in the questionnaire was higher in the intervention (77%) than in the control group (52%). Basic and advanced knowledge regarding immunization was higher (*P* < 0.001) in the intervention group (Table 6). Changes in scores between the pre- and post-surveys for each component of attitudes and beliefs did not differ significantly between the two groups (Table 6). Health literacy regarding immunization was higher (*P* < 0.01) in the intervention group than in the control group (Table 6). Additional file 1: Table S5 shows the internal consistency findings (Cronbach α) for knowledge, attitudes and beliefs scores.

**Table 5:** Intention to receive vaccines after intervention

Outcome	Intervention (*n* = 87)	Control (*n* = 83)	*P*-value
Intention to receive vaccines, n (%)			
No intention	0 (0%)	0 (0%)	
Undecided	1 (1%)	1 (1%)	
Yes, for certain vaccines	19 (22%)	39 (47%)	
Yes, for most vaccines	67 (77%)	43 (52%)	0.001^**^

Fisher’s exact test

^**^p < 0.01

**Table 6:** Knowledge, attitudes and beliefs, and health literacy pre- and post-study

Outcome	Range	Pre-study	Post-study	Pre-study	Post-study	Change from pre-study to post-study		
		Intervention (*n* = 87)		Control (*n* = 84)		Intervention (*n* = 87)	Control (*n* = 84)	*P*-value
Knowledge, mean (SD)								
Basic knowledge	10–30	21.7 (4.0)	27.2 (2.3)	21.7 (4.4)	24.8 (3.0)	5.5 (3.6)	3.0 (3.8)	< 0.001^***^
Advanced knowledge	5–15	6.8 (2.2)	11.9 (2.3)	6.9 (2.2)	9.7 (2.1)	5.1 (2.4)	2.8 (2.5)	< 0.001^***^
Attitudes and beliefs, mean (SD)								
Perceived severity (HBM)	5–15	7.4 (1.6)	8.5 (1.5)	7.6 (1.4)	8.4 (1.4)	1.1 (1.8)	0.8 (1.7)	0.162
Perceived susceptibility (HBM)	1–5	1.8 (1.0)	1.8 (1.0)	1.9 (1.0)	2.0 (1.1)	0.01 (1.1)	0.2 (1.2)	0.204
Perceived benefits (HBM)	4–20	10.2 (3.7)	10.2 (4.2)	10.4 (3.8)	10.2 (4.1)	0.02 (3.5)	−0.3 (3.9)	0.477
Perceived barriers (HBM)	5–25	13.3 (3.2)	14.2 (3.2)	12.3 (3.4)	13.4 (3.6)	1.0 (3.4)	1.1 (3.2)	0.963
Self-efficacy (HBM)	2–10	7.6 (1.5)	7.7 (1.3)	7.7 (1.3)	7.4 (1.3)	0.07 (1.5)	−0.2 (1.4)	0.338
Perceived behavioral control (IBM)	1–5	3.5 (1.2)	3.9 (1.2)	3.5 (1.0)	3.8 (1.1)	0.4 (1.3)	0.3 (1.2)	0.702
Social norm (injunctive)	4–20	16.4 (2.8)	17.5 (2.4)	16.1 (2.4)	17.0 (2.3)	1.1 (2.7)	0.8 (2.7)	0.504
Social norm (descriptive)	2–10	8.2 (2.0)	9.0 (1.5)	8.2 (2.0)	8.7 (1.7)	0.8 (2.1)	0.5 (2.1)	0.739
Health literacy, mean (SD)	1–5	3.3 (0.8)	3.7 (0.7)	3.4 (0.6)	3.6 (0.6)	0.5 (0.8)	0.2 (0.6)	0.009^**^

Mann-Whitney U test

*SD* standard deviation, *HBM* health belief model, *IBM* integrated behavioral model

^**^p < 0.01, ^***^p < 0.001

## Discussion

This study shows the effectiveness of an immunization education program designed to meet mothers’ needs. To our knowledge, this is the first study to demonstrate the effectiveness of an education program for improving the rate of shared parental decision-making regarding the vaccination of their child and health literacy regarding immunization. Overall, the participants in this study were representative of the average Japanese population, with participants earning average incomes and most being college graduates, suggesting that our findings may be applicable to the general Japanese population.

Immunization status for the HBV vaccine was higher in the intervention group than in the control group. A study on the psychosocial determinants of parental intention to vaccinate their newborn child against HBV reported that emphasizing the benefits of vaccination and the child’s risk of hepatitis B infection are important [36]. Our study also emphasized these points, based on the Health Belief Model [28], indicating the effectiveness of our educational program. Immunization status for the rotavirus vaccine was also higher in the intervention than in the control group. A previous Canadian observational study [37] reported that parents who decided before routine immunization to vaccinate their child against rotavirus had previously consulted a doctor or nurse who recommended the vaccine. Given the limited window for rotavirus vaccination, early educational intervention by healthcare professionals might positively impact the immunization status of infants. Intent among parents to immunize their child is a clear predictor of reliable vaccination [36, 37]. Our findings therefore strongly suggest a high actual vaccination rate in the coming months.

Basic and advanced knowledge of immunizations was higher in the intervention group, consistent with previous studies, indicating the effects of early intervention in a prenatal care setting [16, 17].

Health literacy regarding immunization was higher in the intervention group than in the control group. Previous studies have stressed the importance of prenatal education of mothers regarding where to obtain further information on maintaining their child’s health and how to make informed decisions, since prenatal education cannot cover all the information required [38, 39]. In addition, our findings demonstrate that an educational program based on easy access to up-to-date information on immunization for each expectant parent improves health literacy about childhood vaccination. As immunization continues throughout life, the health literacy of parents has a lasting effect on the immunization rates of children. Further, as circumstances surrounding immunization constantly fluctuate, the health literacy of parents helps ensure adequate immunization coverage [40]. Given that improved health literacy leads to healthier behavior [41, 42], our results about health literacy suggest that educational intervention leads to future vaccination.

Regarding the degree of the fathers’ involvement in intervention and decision-making, both parents usually shared the decision-making process even when they could not participate in the intervention during late pregnancy. This suggests a beneficial effect of indirect support provided by the guidebook, which functioned as a communication tool. In both groups, most participants answered, “I think I can discuss vaccinating our child with my child’s father.” Due to a possible ceiling effect, careful examination of parental discussion is required before making any conclusions.

The moderate correlation between parental discussion and decision-making regarding vaccination may suggest the two are linked. Our findings support the effectiveness of an immunization education program that works on fathers both directly and indirectly to help share information on immunization and decision-making on whether to immunize their child. Professionals can help fathers attending perinatal checkups to support the parturient and their infant by providing information on immunization and example discussion points, and illustrating the discussion process when making decisions using the guidebook. If fathers do not attend these checkups, we encourage use of a guidebook as a communication tool to share information with fathers and aid discussions regarding vaccinations. These steps will help facilitate informed decision-making by both parents.

This immunization education program based on mothers’ needs was effective for several reasons. The main reason was that immunization education was sufficient for involving fathers in shared decision-making on optional childhood vaccinations. The absence of significant differences in vaccination status for routine Hib and PCV13 vaccines between the groups might be because timely vaccinations were completed, as participants received the group guidance provided by the hospital. The provision of education programs in other hospitals should therefore include information on the timeliness of vaccinations.

The lack of significant differences in the proportion of parents seeking a pediatrician or primary care physician to administer vaccinations may be due to the hospital having a pediatrics department. Parents could therefore prepare for vaccinations in advance, without having to search for a provider. Consistent with previous findings [16], attitudes and beliefs did not change significantly between the two groups, which is likely because attitudes and beliefs are comparatively stable and remain unchanged [43, 44].

This study contributes to improving immunization rates and the development of support programs for childhood immunization for parents. The education program for expectant parents increases health literacy regarding immunizations and encourages parturients and their partners to share information and engage in joint decision-making about whether or not to immunize their child. Improved immunization rates can reduce the incidence of VPDs. Cost is a major barrier, but this intervention helped parents to obtain knowledge (visualize the burden of vaccination when suffering from a VPD, the benefit and burden of vaccination), and helped both parents to discuss, make decisions and take concrete actions.

Several limitations of our study warrant mention. First, immunization policies and practices vary between countries, and our results might not be generalizable. Further, this study was conducted in a hospital which had already made efforts to improve immunization rates. The vaccination rates of both groups were therefore higher than previously reported [16] and should be carefully interpreted. Second, self-reporting bias needs to be considered [45]. All survey forms, including those examining vaccination status, were self-reported, and the investigators did not confirm participants’ immunization history with charts or maternal and child health record books. Third, it would be preferable if the roles of the intervention educator and researcher were not filled by the same person. Fourth, the cost-effectiveness of the intervention method adopted for group education warrants investigation. Finally, the reliability and validity of the measure of attitude and belief [16] was undergoing verification when the survey was conducted. A measure with validated reliability and validity would be preferable.

## Conclusions

Education for expectant parents increased immunization rates for HBV and rotavirus among children, the number of parents intending to vaccinate their children, knowledge regarding immunization, rate of decision-making by both parents instead of the mother alone, and health literacy regarding immunization. We thus confirmed the effectiveness of an immunization education program. Unique and effective aspects of our educational program were that its interventions encouraged fathers both directly and indirectly to support shared decision-making, based on mothers’ needs, and individualized interventions gave parents access to up-to-date information.

## Supplementary information

**Additional file 1: Figure S1.** Outline of the study. **Table S1.** Questions for basic knowledge related to vaccination. **Table S2.** Questions for advanced knowledge related to vaccination. **Table S3.** Questions for health literacy regarding vaccination of children. **Table S4.** Attitudes and beliefs about vaccine-preventable diseases and vaccination. **Table S5.** Internal consistency (Cronbach α) for knowledge, attitudes and beliefs scores.

## Data Availability

Because the explanatory document for research participants and the research ethics committee did not indicate that data will be released on the internet, the data will not be released or shared. However, data can be obtained from the researchers upon request.

## Abbreviations

HBM: Health Belief Model
HBV: Hepatitis B virus
Hib: *Haemophilus influenzae* type b
IBM: Integrated Behavioral Model
PCV13: 13-valent pneumococcal conjugate vaccine
VPD: Vaccine-preventable disease
