# Supplementary material for: A childhood immunization education program for parents delivered during late pregnancy and one-month postpartum: a randomized controlled trial
Source: BMC Health Serv Res. 2019 Nov 5;19:798. doi: 10.1186/s12913-019-4622-z (PMC6833181; doi:10.1186/s12913-019-4622-z)
Supplement: Supplementary file 1 — Additional file 1: Figure S1. Outline of the study. Table S1. Questions for basic knowledge related to vaccination. Table S2. Questions for advanced knowledge related to vaccination. Table S3. Questions for health literacy regarding vaccination of children. Table S4. Attitudes and beliefs about vaccine-preventable diseases and vaccination. Table S5. Internal consistency (Cronbach α) for knowledge, attitudes and beliefs scores. [file 12913_2019_4622_MOESM1_ESM.pptx]

## Slide 1
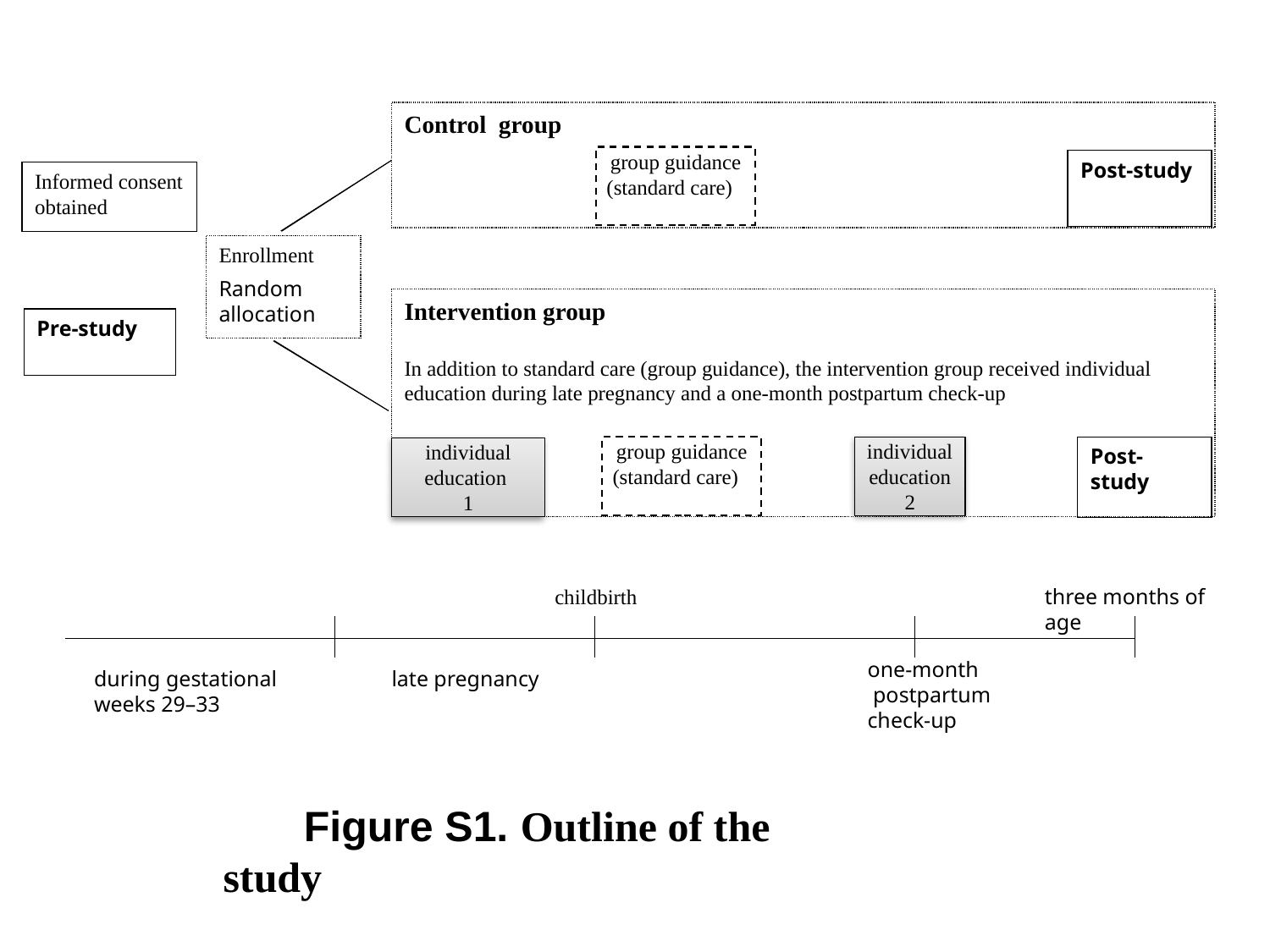

Control group
group guidance
(standard care)
Post-study
Informed consent obtained
Enrollment
Random allocation
Intervention group
In addition to standard care (group guidance), the intervention group received individual education during late pregnancy and a one-month postpartum check-up
Pre-study
group guidance
(standard care)
individual education
2
Post-study
individual education
1
three months of age
childbirth
one-month
 postpartum
check-up
during gestational weeks 29–33
late pregnancy
Figure S1. Outline of the study

## Slide 2
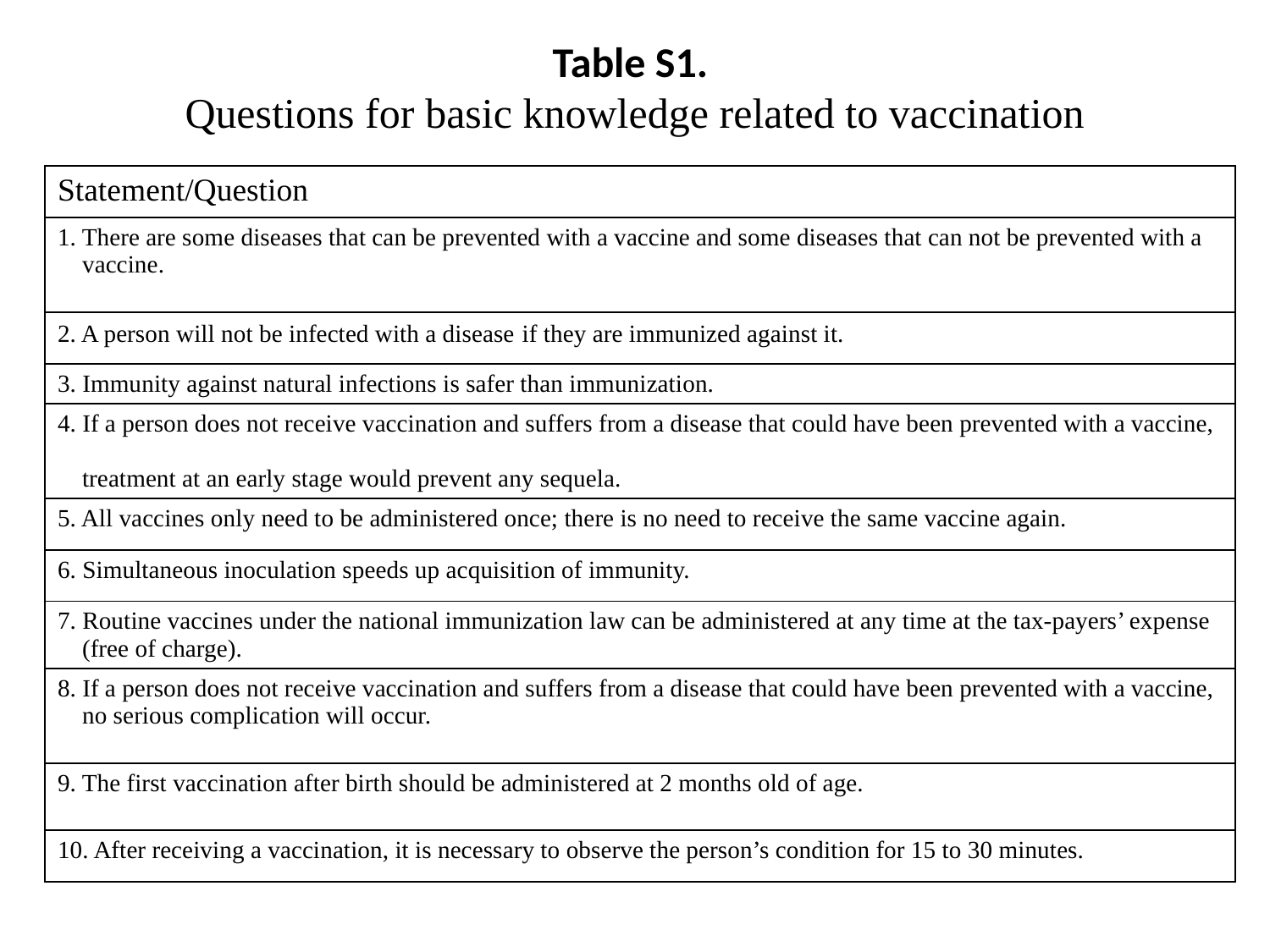

# Table S1.  Questions for basic knowledge related to vaccination
| Statement/Question |
| --- |
| 1. There are some diseases that can be prevented with a vaccine and some diseases that can not be prevented with a vaccine. |
| 2. A person will not be infected with a disease if they are immunized against it. |
| 3. Immunity against natural infections is safer than immunization. |
| 4. If a person does not receive vaccination and suffers from a disease that could have been prevented with a vaccine, treatment at an early stage would prevent any sequela. |
| 5. All vaccines only need to be administered once; there is no need to receive the same vaccine again. |
| 6. Simultaneous inoculation speeds up acquisition of immunity. |
| 7. Routine vaccines under the national immunization law can be administered at any time at the tax-payers’ expense (free of charge). |
| 8. If a person does not receive vaccination and suffers from a disease that could have been prevented with a vaccine, no serious complication will occur. |
| 9. The first vaccination after birth should be administered at 2 months old of age. |
| 10. After receiving a vaccination, it is necessary to observe the person’s condition for 15 to 30 minutes. |

## Slide 3
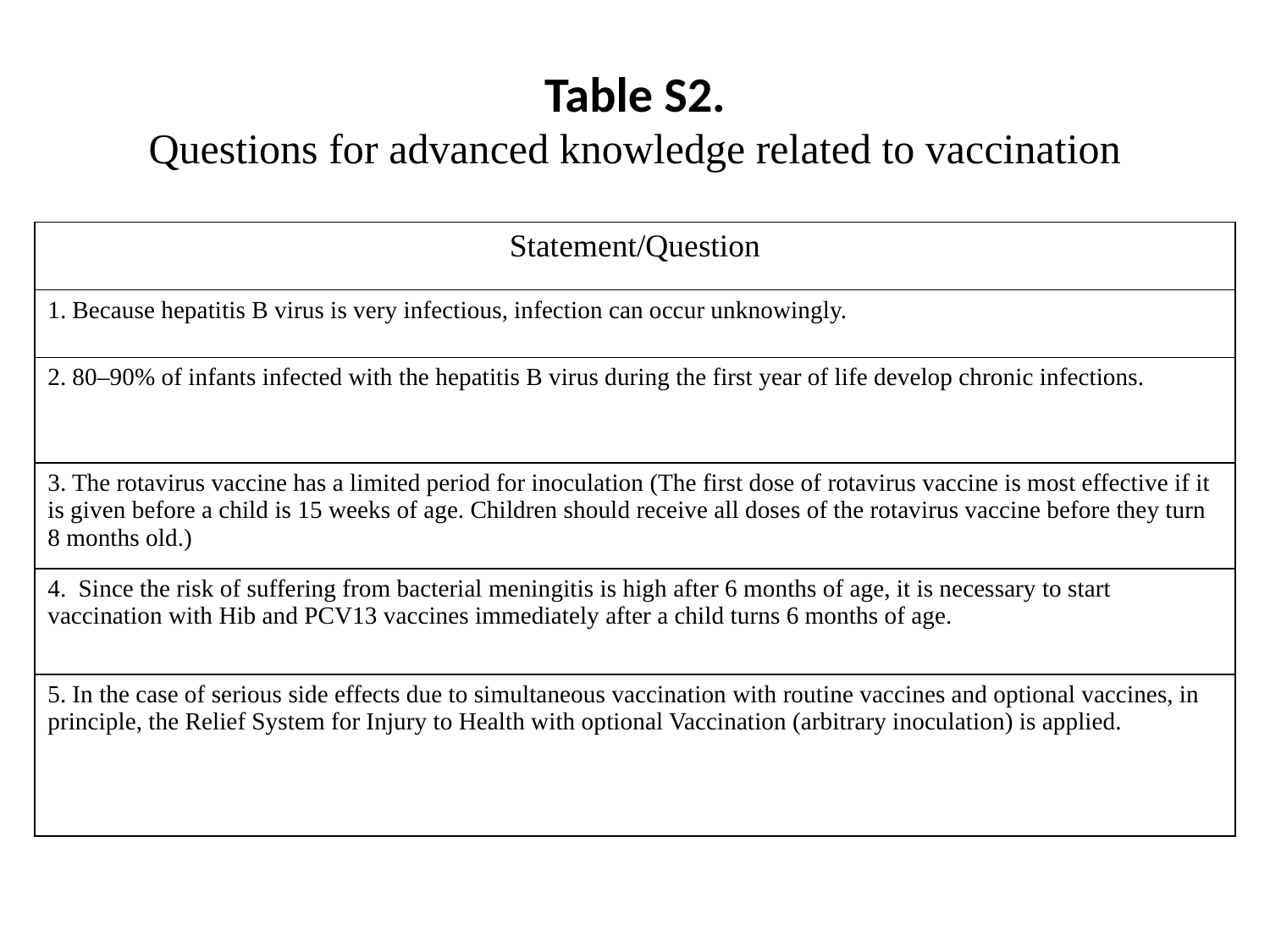

# Table S2. Questions for advanced knowledge related to vaccination
| Statement/Question |
| --- |
| 1. Because hepatitis B virus is very infectious, infection can occur unknowingly. |
| 2. 80–90% of infants infected with the hepatitis B virus during the first year of life develop chronic infections. |
| 3. The rotavirus vaccine has a limited period for inoculation (The first dose of rotavirus vaccine is most effective if it is given before a child is 15 weeks of age. Children should receive all doses of the rotavirus vaccine before they turn 8 months old.) |
| 4.  Since the risk of suffering from bacterial meningitis is high after 6 months of age, it is necessary to start vaccination with Hib and PCV13 vaccines immediately after a child turns 6 months of age. |
| 5. In the case of serious side effects due to simultaneous vaccination with routine vaccines and optional vaccines, in principle, the Relief System for Injury to Health with optional Vaccination (arbitrary inoculation) is applied. |

## Slide 4
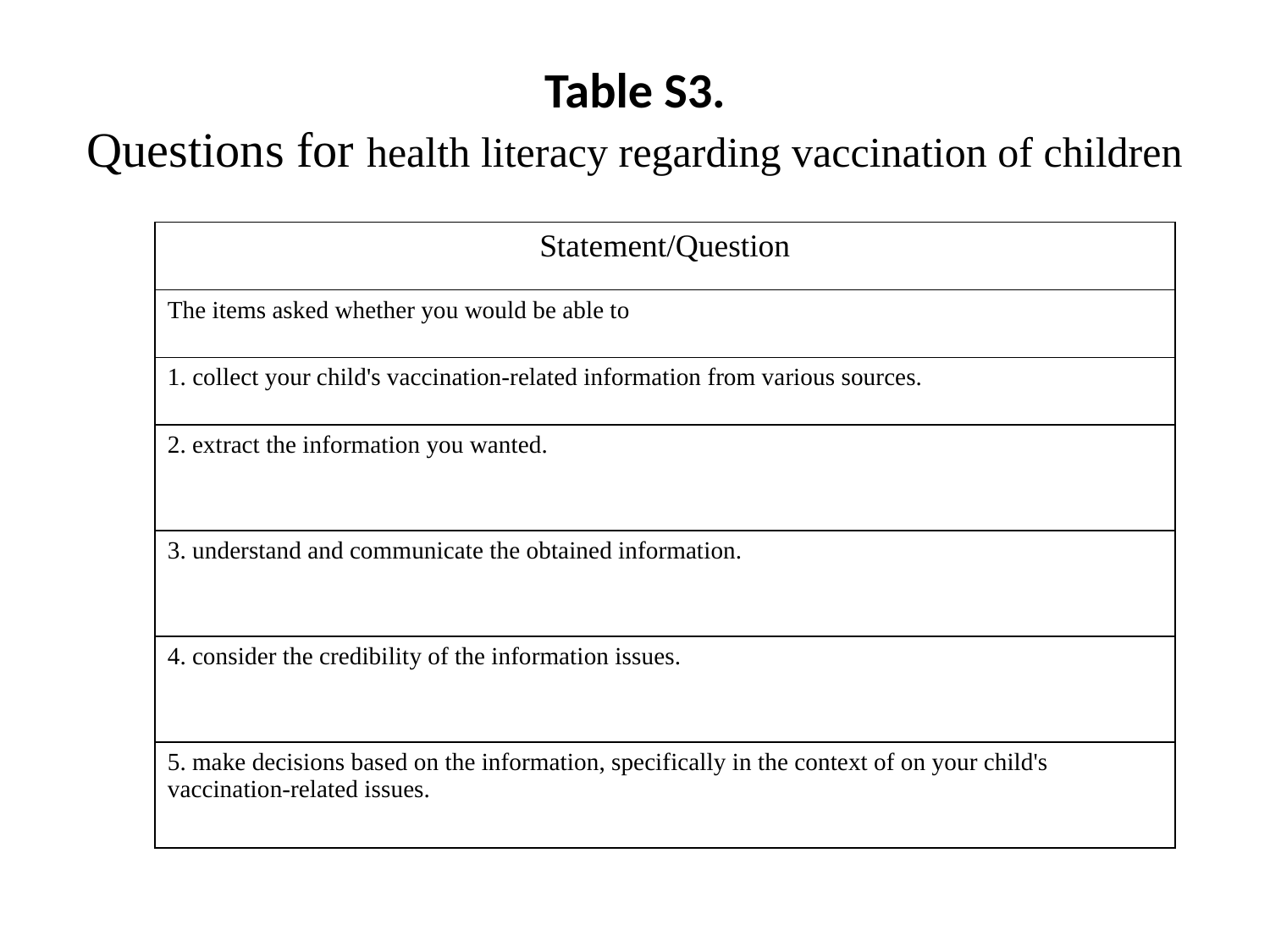

# Table S3. Questions for health literacy regarding vaccination of children
| Statement/Question |
| --- |
| The items asked whether you would be able to |
| 1. collect your child's vaccination-related information from various sources. |
| 2. extract the information you wanted. |
| 3. understand and communicate the obtained information. |
| 4. consider the credibility of the information issues. |
| 5. make decisions based on the information, specifically in the context of on your child's vaccination-related issues. |

## Slide 5
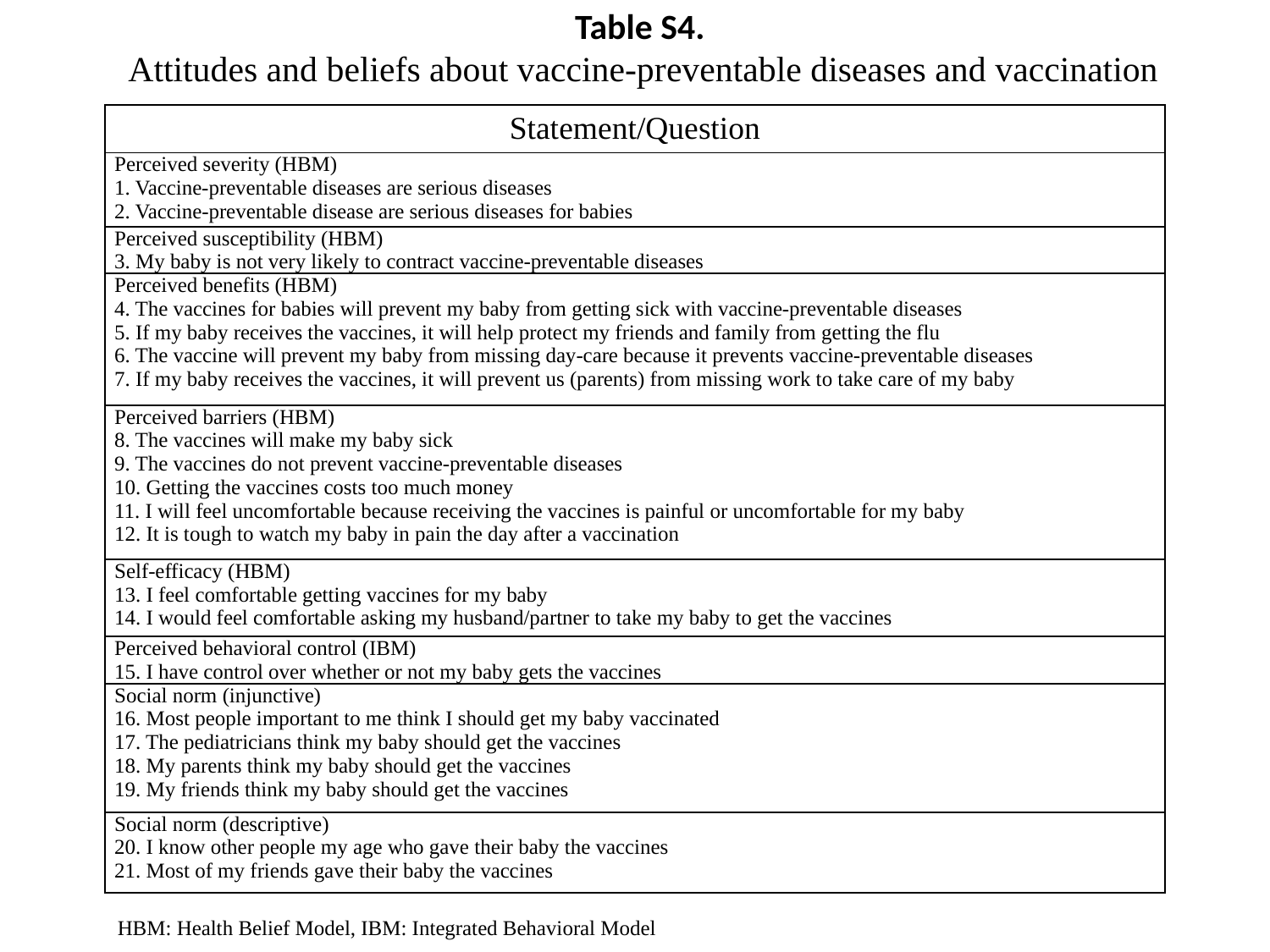

# Table S4. Attitudes and beliefs about vaccine-preventable diseases and vaccination
| Statement/Question |
| --- |
| Perceived severity (HBM) 1. Vaccine-preventable diseases are serious diseases 2. Vaccine-preventable disease are serious diseases for babies |
| Perceived susceptibility (HBM) 3. My baby is not very likely to contract vaccine-preventable diseases |
| Perceived benefits (HBM) 4. The vaccines for babies will prevent my baby from getting sick with vaccine-preventable diseases 5. If my baby receives the vaccines, it will help protect my friends and family from getting the flu 6. The vaccine will prevent my baby from missing day-care because it prevents vaccine-preventable diseases 7. If my baby receives the vaccines, it will prevent us (parents) from missing work to take care of my baby |
| Perceived barriers (HBM) 8. The vaccines will make my baby sick 9. The vaccines do not prevent vaccine-preventable diseases 10. Getting the vaccines costs too much money 11. I will feel uncomfortable because receiving the vaccines is painful or uncomfortable for my baby 12. It is tough to watch my baby in pain the day after a vaccination |
| Self-efficacy (HBM) 13. I feel comfortable getting vaccines for my baby 14. I would feel comfortable asking my husband/partner to take my baby to get the vaccines |
| Perceived behavioral control (IBM) 15. I have control over whether or not my baby gets the vaccines |
| Social norm (injunctive) 16. Most people important to me think I should get my baby vaccinated 17. The pediatricians think my baby should get the vaccines 18. My parents think my baby should get the vaccines 19. My friends think my baby should get the vaccines |
| Social norm (descriptive) 20. I know other people my age who gave their baby the vaccines 21. Most of my friends gave their baby the vaccines |
HBM: Health Belief Model, IBM: Integrated Behavioral Model

## Slide 6
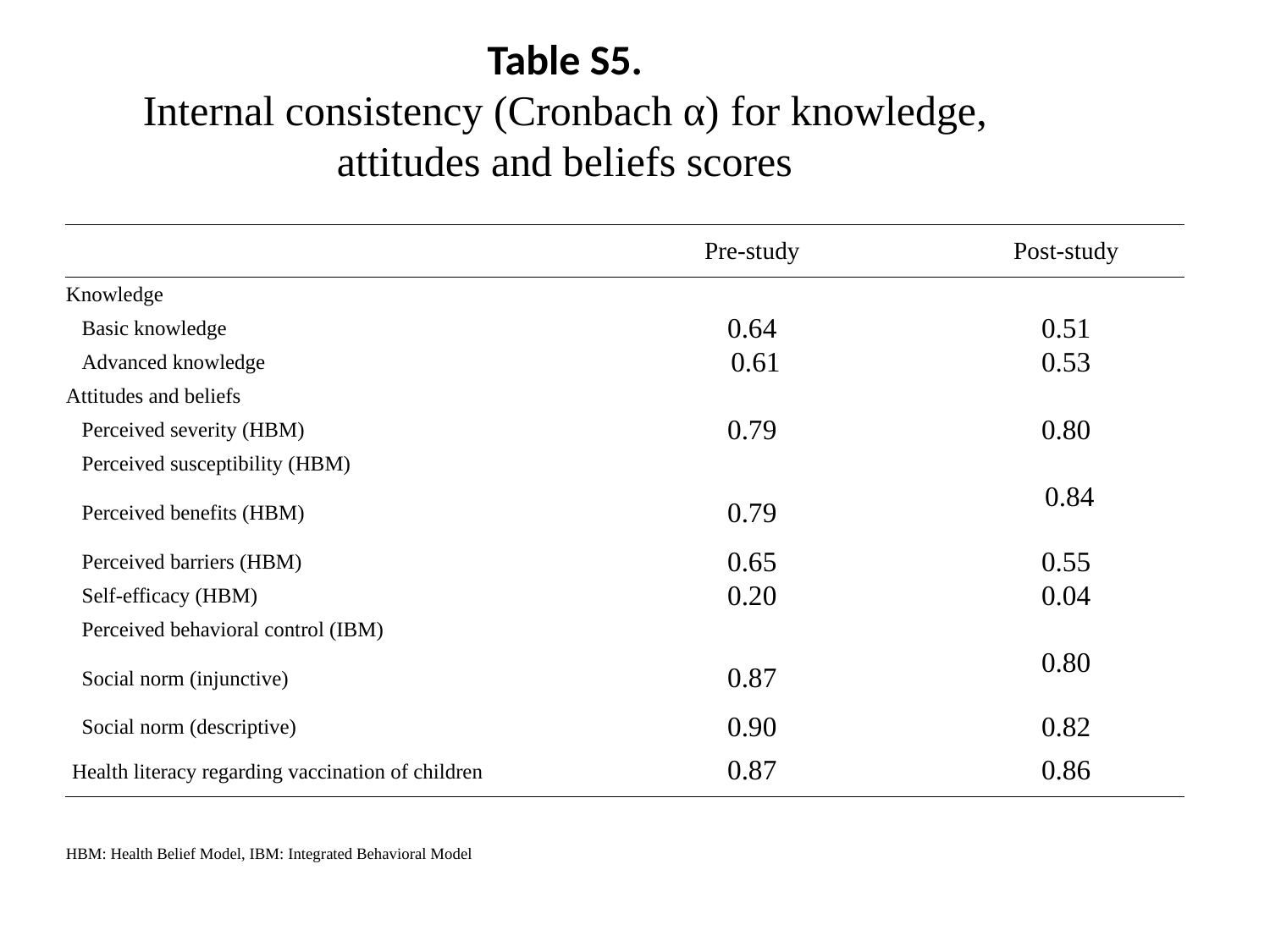

# Table S5. Internal consistency (Cronbach α) for knowledge, attitudes and beliefs scores
| | | | | | | |
| --- | --- | --- | --- | --- | --- | --- |
| | Pre-study | | | | | Post-study |
| | | | | | | |
| Knowledge | | | | | | |
| Basic knowledge | 0.64 | | | | | 0.51 |
| Advanced knowledge | 0.61 | | | | | 0.53 |
| Attitudes and beliefs | | | | | | |
| Perceived severity (HBM) | 0.79 | | | | | 0.80 |
| Perceived susceptibility (HBM) | | | | | | |
| Perceived benefits (HBM) | 0.79 | | | | | 0.84 |
| Perceived barriers (HBM) | 0.65 | | | | | 0.55 |
| Self-efficacy (HBM) | 0.20 | | | | | 0.04 |
| Perceived behavioral control (IBM) | | | | | | |
| Social norm (injunctive) | 0.87 | | | | | 0.80 |
| Social norm (descriptive) | 0.90 | | | | | 0.82 |
| Health literacy regarding vaccination of children | 0.87 | | | | | 0.86 |
| | | | | | | |
| HBM: Health Belief Model, IBM: Integrated Behavioral Model | | | | | | |
